# Surrogate-assisted optimization of roll-to-roll slot die coating

**DOI:** 10.1038/s41598-025-11279-1

**Published:** 2025-08-09

**Authors:** Christopher Passmore, Kai E. Wu, Jonathan R. Howse, George Panoutsos, Stephen J. Ebbens

**Affiliations:** 1 School of Chemical Materials and Biological Engineering, The University of Sheffield, S1 3JD Sheffield, UK; 2 School of Electrical and Electronic Engineering, The University of Sheffield, S1 4WD Sheffield, UK

**Keywords:** Slot die coating, Machine learning, Optimization, Coating uniformity, Chemical engineering, Surface chemistry, Chemical engineering, Design, synthesis and processing, Computational methods

## Abstract

Roll-to-roll slot die coating is a key wet processing technique, where achieving a specific thickness with minimal variability is crucial. However, the numerous input parameters make optimization complex. Despite its advanced applications, computer-aided optimization remains underutilized, leaving potential performance improvements untapped. Due to the lack of accurate first-principle models, machine learning offers a promising approach. This study employs Radial Basis Function Neural Networks as surrogate models trained on experimental data to optimize roll-to-roll slot die coating. These models predict coating thickness and uniformity with mean absolute errors below 11.5 %. Key process parameters are identified, with shim thickness and substrate velocity having the greatest impact on coating uniformity, while coating gap played a lesser role. An evolutionary optimization approach identified new operating parameters, leading to improved coating properties. Experimentally, these optimized conditions achieved the five lowest recorded uniformity values and increased the hyper-volume fraction from 0.68 to 0.84. Some prediction inconsistencies were observed, likely due to the high sensitivity of lab-scale equipment, which is expected to improve at an industrial scale. This work paves the way for wider adoption of machine learning and accurate metrology in slot die coating.

**Supplementary Information:**

The online version contains supplementary material available at 10.1038/s41598-025-11279-1.

## Introduction

Thin films with a high technical specification have many applications, including within lithium-ion batteries^1,2^, solar panels^3^ and polymer electrolyte membrane fuel cells^4,5^. Roll-to-roll slot die coating is a widely used technology for the industrial scale manufacture of thin films, which involves pumping a fluid through a slot in a metal block onto a moving substrate^4,6^. The high line speed, high material utilization and ability to pre-select coating thickness are factors which have contributed to roll-to-roll slot die coating becoming ubiquitous in state-of-the-art thin film manufacture^7^.

**Fig. 1:**
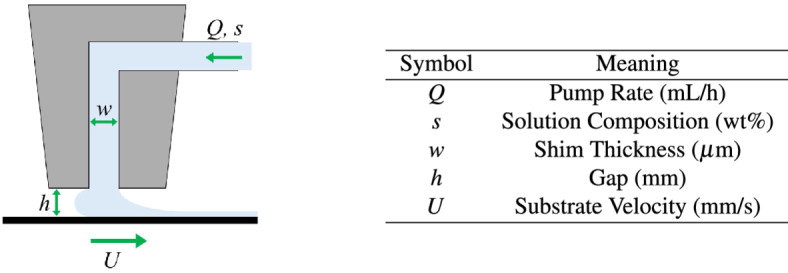
Schematic of the side view of slot die, labelled with slot die coating process parameters.

Slot die coating has many adjustable process parameters, which influence the coating formed. These parameters, as illustrated in Fig. 1, include substrate velocity, coating gap, shim thickness and composition of coating solution^4,8^. Slot die coating forms defect-free coatings using parameter sets within the operating window^4^. Outside of this window, the coating process is susceptible to significant defects such as ribbing, dripping and air entrapment. However, even when remaining within the operating window, different sets of coating parameters give different coating properties^9,10^. The wet coating thickness, for example, can vary within the operating window depending on the ratio of pump rate to substrate velocity^4^. However, more subtle features such as coating uniformity and edge quality also depend on the process conditions used^10,11^. Schmitt et al. termed regions within the operating window with a high coating uniformity, the quality window^10^.

Many theoretical models can predict the operating window for slot die coating such as those by Ruschak^12^, Higgins and Scriven^13^ and Yamamura^14^. However, to the best of the authors’ knowledge, there is no efficient theoretical understanding or analytical models, of how process parameters affect features of the coating *within* the operating window. This is noteworthy as coating properties such as unexpected coating thickness deviations and coating uniformity have a large influence on the performance of subsequent devices^15^. For example, a high coating uniformity is essential for Li-ion battery electrodes as it minimizes rejection rate and has a large influence on the electrochemical performance of the electrode^1,16^. As a specific example of this, Mohanty *et al.* found that non-uniform Li-ion NMC electrode coatings gave poor rate capability, especially at higher rates, and a lower Coulombic efficiency^17^. Coating uniformity is also an important coating feature for organic photovoltaic devices, with a higher uniformity resulting in improved device performance^18^.

Despite the dogma that slot die coating provides perfect thickness control, in reality small changes in the coating width can occur depending on the process parameters used, which in turn alters the coating thickness^19^. This effect is due to the non-Newtonian behavior of polymeric coating solutions^20^. The coating’s thickness determines the energy density of a Li-ion battery, with a thinner than expected electrode giving a lower cell energy density^21^. Additionally, a thicker than expected electrode may lead to mass transport limiting charging/discharging rates. These differences in coating thickness are particularly significant for industrial operators in applications which utilize stripe coatings or with strict thickness requirements.

The lack of theoretical modeling and understanding of how input parameters impact these fundamental coating properties in slot die coating^4^ means that operators currently optimize production through iterative, trial and error adjustments^1,11^. The large amount of process parameters and competing outputs exacerbate the complexity of this optimization and make trial and error unlikely to result in the coating process being truly optimized. This has cost implications, due to the waste material produced and production time lost during this laborious process. Additionally, this type of optimization leaves potential device performance improvements untapped.

Despite computer aided optimization having been implemented in a wide variety of manufacturing processes in other sectors, such as additive manufacturing^22,23^ and pharmaceutical manufacturing^24^, it is not routinely applied in industrial roll-to-roll slot die coating lines. Harnessing computer-aided optimization in this context could unlock significant cost and performance benefits for a wide range of devices. Furthermore, such an approach has the potential to provide valuable insights into the relationship between key coating parameters and the resultant properties of the coating.

The literature has documented some instances of computer-assisted optimization for roll-to-roll slot die coating^16,25,26^. However, there are no reports linking fundamental process parameters to critical coating properties, highlighting a significant gap in the understanding of slot die coating within its operating window. Additionally, there has not been any utilization of a machine learning model to provide experimental improvements in coating features. This disconnect highlights a divide between the theoretical modeling and practical application, raising concerns about the effectiveness or applicability of the previously reported methods.

Machine learning-based surrogate optimization is well-suited for slot die coating, due to the process’s complexity and the numerous interacting inputs and outputs. Surrogate modeling involves constructing computationally efficient approximations of approximate complex, computationally expensive, or time-intensive simulations using experimental data and is well known for capturing intricate non-linear relationships^16,27^. Surrogate models are particularly effective when analytical models are unavailable.

There are a range of analytical approaches and multivariate modeling tools that can be used for modeling industrial processes. For instance, multiple linear regression (MLR), polynomial regression (PR)^28^, principal component analysis (PCA), latent variable model (LVM), orthogonal partial least squares (OPLS)^29^, Gaussian process (GP), and Artificial Neural Networks (ANN)^30^. Among these, Radial Basis Function Neural Networks (RBFNNs), are a notable machine learning modeling method due to their ability to accurately describe complex nonlinear relationships while maintaining computational efficiency. With its universal approximation capability^31^, RBFNNs excel at modeling complex systems with high accuracy and efficiently capturing local variations in data.

However, alternative surrogate modelling methodologies also warrant consideration. For example, Gaussian Process regression is widely recognized for its flexibility and ability to provide uncertainty estimates, making it a strong candidate for problems where quantifying prediction confidence is important. Similarly, Support Vector Regression, which adapts support vector machine methodology to regression tasks, is known for its robustness in high-dimensional spaces and often achieves good generalization on relatively small datasets^32^. Comparative studies have demonstrated that while RBFNNs typically offer fast training and effective local modelling, Gaussian Process models may outperform them in terms of predictive error under certain circumstances, particularly with small datasets or when uncertainty quantification is required. Furthermore, recent research has shown that SVR can deliver competitive performance, though its accuracy might be surpassed by RBFNNs and Gaussian Process models under some conditions, such as in delamination detection in composite laminates^33^. Therefore, incorporating comparative analysis with these surrogate modelling techniques can further reinforce the strengths and appropriate use-cases for RBFNNs, highlighting the need for model selection based on the specific characteristics of the data and the modelling objectives.

Although gradient-based optimization techniques can effectively explore solution spaces, they have inherent limitations such as generalization challenges and a lack of performance guarantees. Surrogate-assisted evolutionary computation addresses these issues by using computationally efficient models to estimate fitness functions in evolutionary algorithms. This approach is particularly valuable for complex optimization problems with computationally expensive objective functions. By employing surrogate models like RBFNNs, this approach significantly reduces the need for costly experimental evaluations, accelerating both exploration and exploitation of the parameter space. Additionally, acquisition functions further balance exploration and exploitation, making this method particularly effective for high-dimensional optimization tasks.

Reference Vector Guided Evolutionary Algorithm (RVEA), a flexible and scalable meta-heuristic evolutionary optimization method^34^, is well-suited for complex processes, that traditional multi-objective problem algorithms struggle with in terms of performance and diversity maintenance^35^. RVEA utilizes a uniform distribution of *reference vectors* - directions in the input parameter space- to guide the search for optimal solutions. This method ensures a diverse set of solutions spread across the Pareto front, which represents the set of optimal trade-offs between conflicting objectives. Additionally, RVEA’s adaptive angle penalty mechanism dynamically adjusts selection pressure, maintaining a balance between convergence and diversity, making it well-suited for complex, high dimensional problems.

This article presents an experimentally validated optimization approach for roll-to-roll slot die coating, combining a RBFNN surrogate model with RVEA. The coating composition used resembles many industrially relevant coatings, such as slurries for lithium ion batteries and solar cells^1,36^, making insights gained applicable across a wide range of fields. This article aims to promote the widespread implementation of machine learning-based optimization in roll-to-roll slot die coating, offering potential improvements in cost, performance and process understanding.

## Methods

### Coating solution preparation

All coating solutions were prepared by adding the appropriate mass of ethanol (32221 Sigma Aldrich) to titanium oxide 21 nm nanopowder (718467 Sigma Aldrich). Polyvinylpyrollidone (PVP360 Sigma Aldrich) was then slowly added to the solution ensuring 1:1 mass ratio of polyvinylpyrrolidone to titanium dioxide. The resultant solution was then stirred overnight. All coating solutions were used within 24 hours of being prepared.

### Roll-to-roll slot die coater

**Fig. 2:**
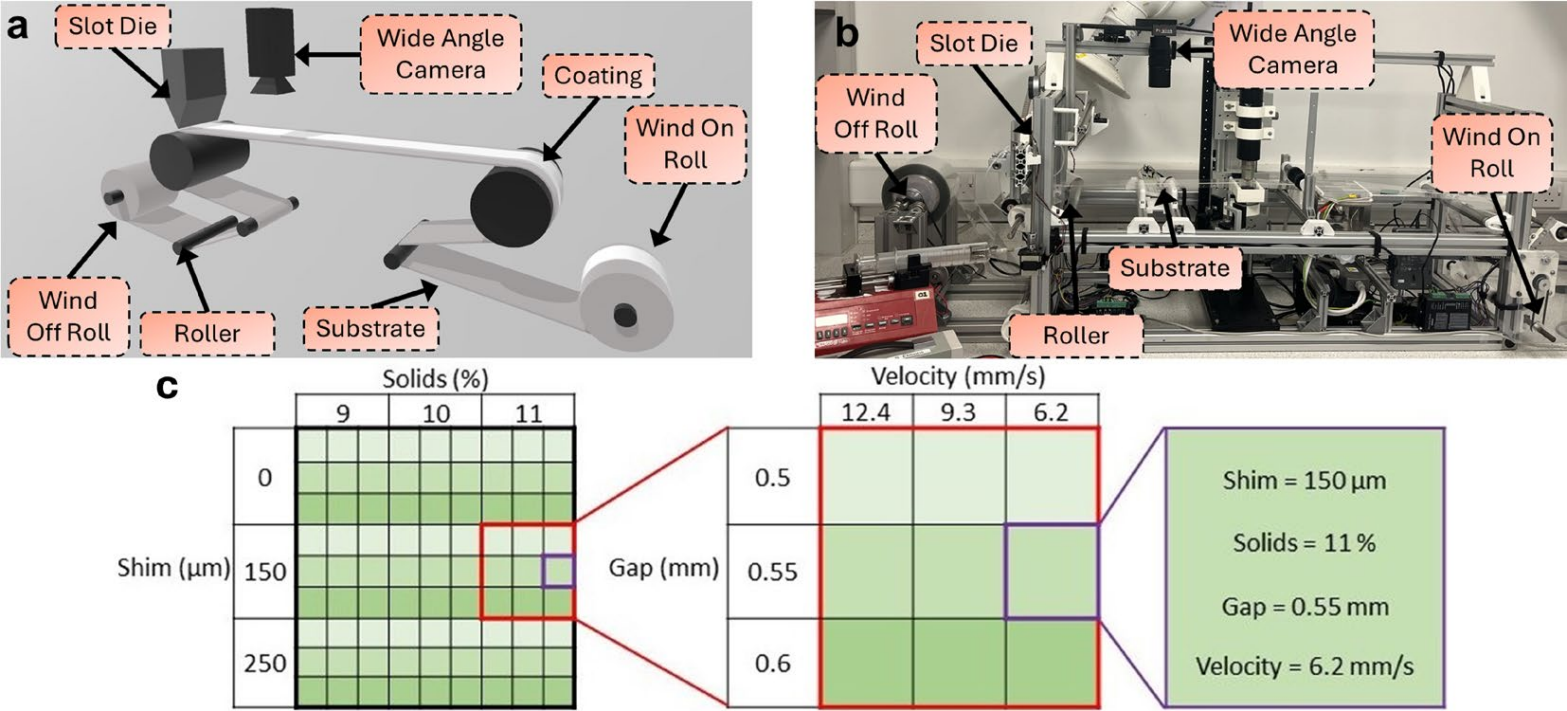
Schematic showing roll-to-roll slot die coater and the position of wide angle camera. Image of roll-to-roll slot die coater with in-line wide angle camera metrology. Schematic showing the matrix of inputs used for the systematic selection of initial experimental data parameters. The red square indicates one experimental trial, using the same solids and shim thickness. The purple square indicates a single parameter set within each trial.

A roll-to-roll slot die coater, equipped with an in-line wide angle transmission camera, was used to collect all data for this article. A schematic and image of the experimental equipment is shown in Fig. 2, and is identical to that previously reported^37^. The camera captures images of the entire coated film, utilizing the substrate velocity and a timing loop to prevent any overlap between consecutive images.

Initial experimental data from the roll-to-roll slot die coater was collected in a full factorial symmetric grid, varying shim thickness, coating solution composition, substrate velocity and coating gap. These inputs are systematically varied, with each input taking a high, medium and low value, resulting in a grid of parameter sets. This approach ensures the input parameter space is well explored and minimizes bias in the data set. This in turn minimizes the risk of leaving significant regions of the parameter space unexplored. This method of data collection also ensures that the effect of individual input parameters on coating properties is captured, improving the model’s ability to predict their effect.

The pump rate was adjusted for each experiment to account for changes in substrate velocity and solids content, to give the same theoretical areal coverage. Therefore, pump rate is dependent on other parameters. For example, if the substrate velocity was halved, the pump rate was also halved. Additionally, if the solids content of the coating was reduced from 10 to 9%, the pump rate was increased by a factor of 10/9. This adjustment is based on the widely reported pre-metering ability of slot die coating to ensure all coatings have the same theoretical dry areal coverage^4,38^. Each parameter set was run for two minutes, to allow time for the coating to reach steady state. Only data from the second minute of each experiment was used and the data from the preconditioning period was discarded. This ensures each data point is high value and contains minimal noise.

In total, 81 parameter sets are collected for the initial experimental data in nine trials, which in turn each contain nine parameter sets as shown in Fig. 2c. Each experimental trial begins with two minutes of preconditioning with a coating gap of 0.50 mm, substrate velocity of 12.4 mm/s and pump rate of 48 mL/hr. After the preconditioning period, the parameter sets which form the initial experimental data are collected.

### Image analysis

**Fig. 3:**
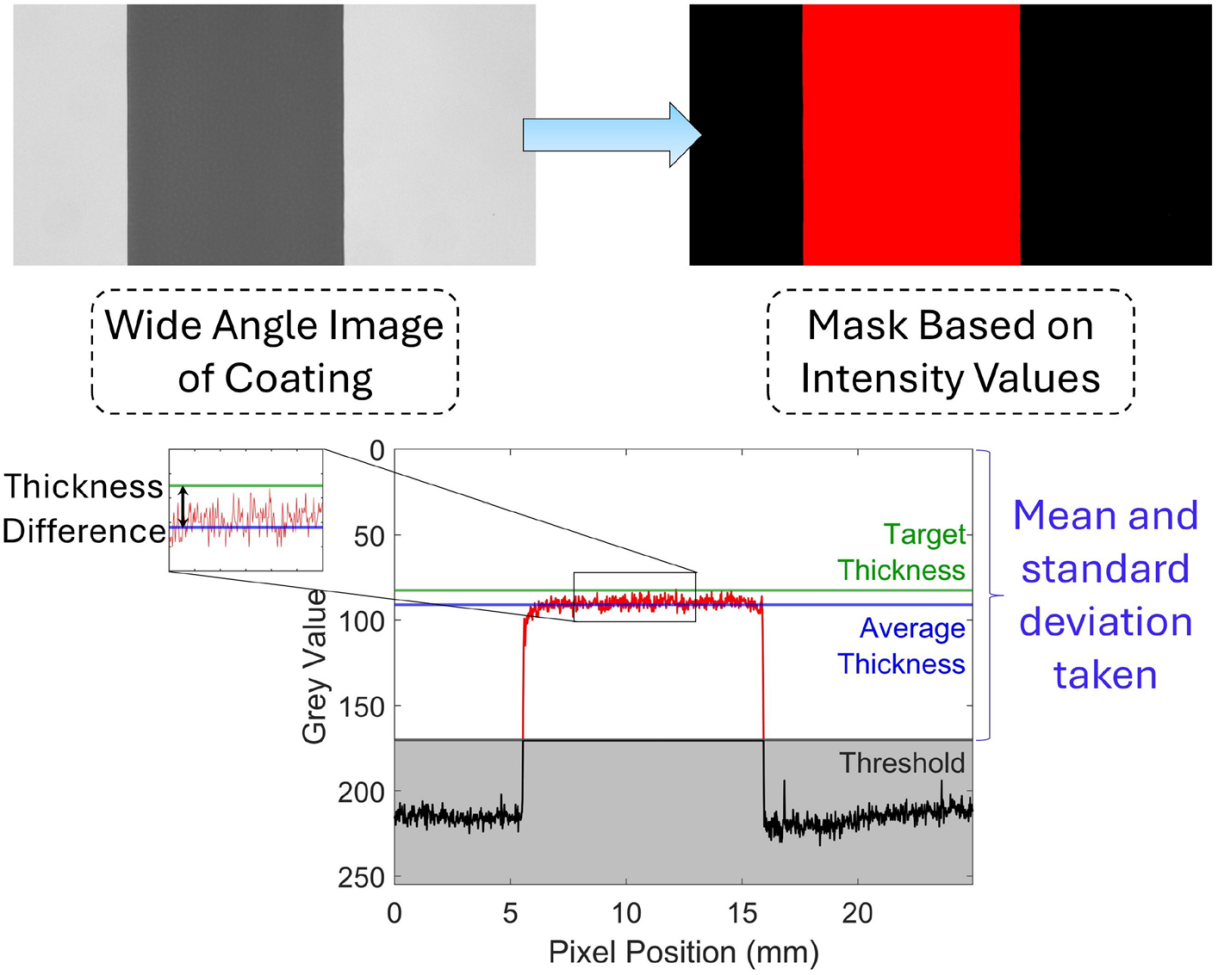
Schematic illustrating the image analysis process; first the green color plane is extracted from the wide angle image of the coating. After that, a threshold is conducted to isolate the coated region from the substrate. The mean grey value and standard deviation of grey values are then calculated from the pixel intensities within the threshold region. The thickness difference is calculated as the difference between the mean grey value and the target thickness. The target thickness is the expected coating thickness assuming a 10 mm wide uniform coating is produced.

Image analysis of the wide angle image is conducted, as shown in Fig. 3. The green color plane is extracted from the image, followed by a threshold to detect the coated region. Since the light source is directly behind the substrate, the intensity recorded by the camera is influenced by both the absorption coefficient and thickness of the coating. The “grey scale” value at each pixel corresponds to the amount of light transmitted through the film with the darker pixels representing a thicker coating and vice versa. The average pixel intensity value in the coated area is recorded as the “mean grey”^39^. The “mean grey” was fitted to an extended Beer-Lambert law using calibration samples to convert to real world thickness estimates (Supplementary Information Fig. S1). The standard deviation of the intensity values is also measured, and used to give standard deviation of wet coating thickness.

By employing in-line metrology to investigate wet coating behavior, the initial experimental data set was efficiently gathered within 3 hours of instrument time. This contrasts to conventional off-line methods, which require relocating regions of the film corresponding to specific parameters and subsequent separate analysis.

### Surrogate modeling

RBFNNs are a type of artificial neural networks that consist of three main layers; input, hidden, and output^40^. The input layer receives the input data, transforms it and passes it onto the hidden layer. In the context of wet coating thickness estimation, this layer captures the absolute thickness measurements and compares them with a target thickness value to compute the thickness difference. The hidden layer is composed of neurons that use radial basis functions as their activation functions. The radial basis function is typically a Gaussian function, though other functions can be used. The Gaussian function is given by;

1 $$\begin{aligned} h(x) = \exp \left( -\frac{\Vert x - c\Vert ^2}{2 \sigma ^2}\right) \end{aligned}$$

Where *x* is the input, *c* is the centre of a radial basis function neuron, $\sigma$ is a width parameter that controls the spread of the function. The hidden layer neurons respond to inputs in relation to their proximity to each neuron’s centre, making RBFNNs particularly effective for pattern recognition and interpolation tasks. Finally, the output layer computes a weighted sum of outcomes from the hidden layer.

The characteristics of RBFNN possess an intrinsic locality and are significantly influenced by the spatial separation between decision space points and the centers of radial basis functions.

Cross validation of the RBFNN was conducted, and the mean absolute error (MAE) and root mean squared error (RMSE) are used to evaluate performance. 60 parameter sets were used as training data for the RBFNN during cross validation with 21 being used to test the data. The result is a model that has the best performance based on how well they predicted the coating properties of the cross-validation data.

### Surrogate-assisted optimization

In this study, RBFNNs are used as the surrogate model and RVEA as the optimization algorithm. To refine the solution space, a stochastic search method was employed, introducing random perturbations to top-performing solutions. This helps prevent convergence to local optima, thereby enhancing the likelihood of discovering the global optimum, or Pareto front. The Pareto front consists of the predicted set of optimal conditions in multi-objective optimization, where no improvements can be made to one coating feature without worsening another. Each point on the Pareto front corresponds to an experimentally viable combination of process parameters offering guidance to operators when selecting new parameters based on limited experimental data.

For the optimization, the RVEA targeted a wet coating thickness of approximately 110 $\upmu$m, and a standard deviation of wet coating thickness of 0 $\upmu$m.

All computational experiments and analyses were performed on a computer with the following specifications: Intel(R) Core(TM) i7-10700 CPU operating at 2.90 GHz, 32 GB of RAM (31.7 GB usable), and a 64-bit Windows operating system.

### Experimental validation

A second round of experiments was conducted to validate the ability of the RBFNN-RVEA method to produce parameter sets which give improved coating properties. The 10 validation points were collected in 10 experiments, each starting with the same two minute preconditioning period as in the initial experimental data. The experimental conditions of the validation data was held for two minutes. Image analysis of the second minute of each validation data was performed using the same method as for the initial experimental data.

## Results and discussion

### Initial experimental data collection

**Fig. 4:**
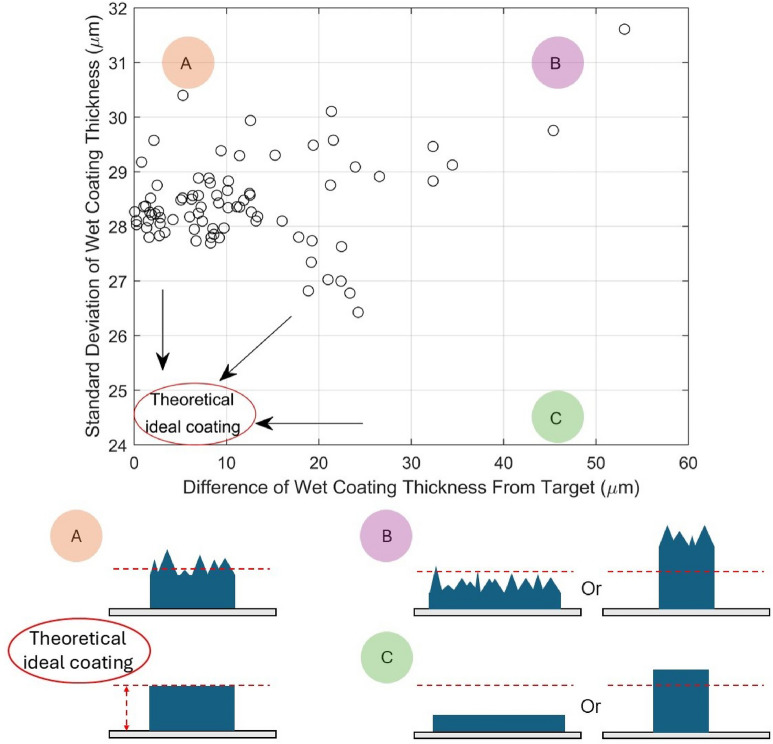
Graph showing difference of wet coating thickness from target and the standard deviation of wet coating thickness for roll-to-roll slot die coating experimental data. The location of a theoretical ideal coating (which may not exist) is indicated by the red ellipse. Region *A* denotes a region with good thickness, bad uniformity, *B* bad thickness, bad uniformity and *C* bad thickness, good uniformity. A schematic illustrating the cross-sections of coatings within these regions is shown below the graph, with the red dashed line indicating the target thickness.

Initial experimental data was collected from the roll-to-roll slot die coater using a coating solution containing a polymeric binder and inorganic particles. In Fig. 4 the effect of coating width changing depending on the coating parameters used is assessed by determining the magnitude of the difference of wet coating thickness from a target value of 110 $\upmu$m. This thickness was chosen as the target due to its relevance to industrial applications. For example, this thickness is similar to the optimal electrode thickness of a lithium-ion battery electrode where cell energy density is maximized before mass transport becomes a limiting factor^21^.

In Fig. 4 coating uniformity is assessed by determining the standard deviation of coating thickness, with a higher standard deviation of wet coating thickness indicating a less uniform coating^41^.

The data in Fig. 4 suggests that there is a trade off between coating uniformity and wet coating thickness difference, hence a Pareto front is expected as Fig. 4 approximately depicts. For example, in the initial experimental data, the only way to achieve a standard deviation of wet coating thickness of less than 27 $\upmu$m is at the expense of high difference of wet coating thickness from target thickness. This demonstrates an intrinsic trade off and competition between these two coating features. To the best of the authors’ knowledge, this is the first time that a compromise between coating uniformity and coating thickness within the operating window has been demonstrated and quantified. This finding shows the dogma of precisely metered coating is challenged even within the operating window.

### RBFNN modeling and RVEA optimization

The initial experimental data was then modeled using a RBFNN, as described in Methods Section. Upon cross-validation, the RBFNN model for difference of wet coating thickness from target value gave a root mean squared error (RMSE) value of 5.9 % for training data and 10.38 % for validation and mean absolute error (MAE) value of 4.24 % for training data and 7.59 % for validation data. The RBFNN model for standard deviation of wet coating thickness gave a RMSE value of 5.39 % for training data and 11.41 % for validation data and MAE of 4.30 % for training data and 8.38 % for validation data. These values confirm the ability to model the roll-to-roll slot die coating system. The total computing time was less than 1 hour. Further modeling was conducted using GP and SVR, but these methods gave worse performance with MAE values of less than 12.6 % and 16.1 % respectively. Further details of unused modeling methods can be found in Supplementary Information.

**Fig. 5:**
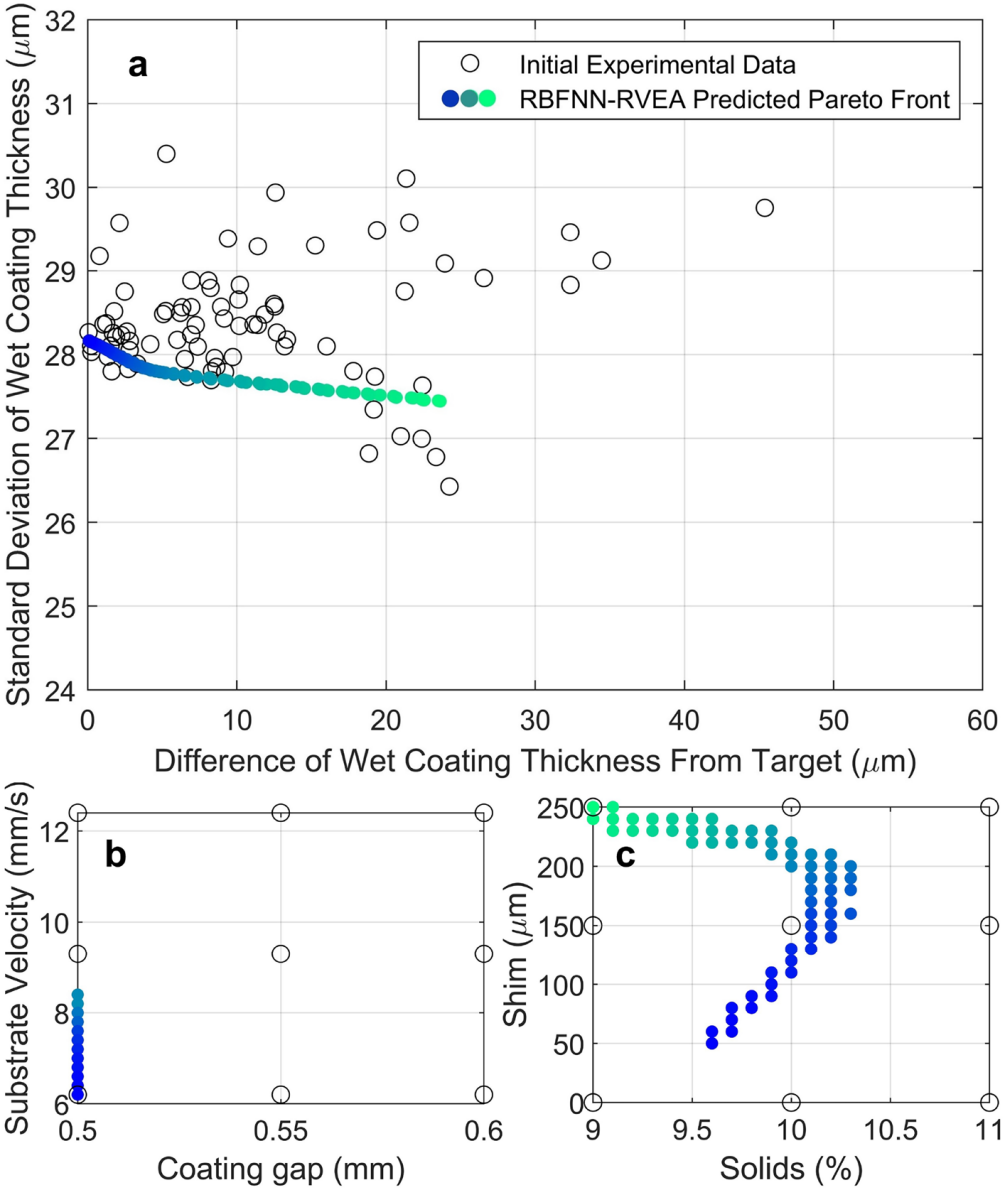
(**a**) Initial experimental data with RBFNN-RVEA predicted Pareto front. The solid circles are the predicted Pareto front and are color coded based on their relative position within the Pareto front to allow cross-referencing with (**b**,**c**). (**b**) Graph showing Pareto front from (**a**) as a function of substrate velocity and coating gap. (**c**) Graph showing Pareto front from (**a**) as a function of shim thickness and coating solution solids. (**b**,**c**) Allow the distribution of the shown models predicted optimal parameters (in colour) to be compared to the original DOE parameter grid (black circles).

Figure 5a shows the RBFNN-RVEA predicted Pareto front plot overlaid on the initial experimental data. The effect of process parameters on coating features can be predicted from the RBFNN model. For example, Fig. 5b,c show how process parameters vary with the position along the predicted Pareto front, with a blue dot indicating a coating with a low difference of wet coating thickness from target and a green dot indicating a high difference of wet coating thickness from target.

**Fig. 6:**
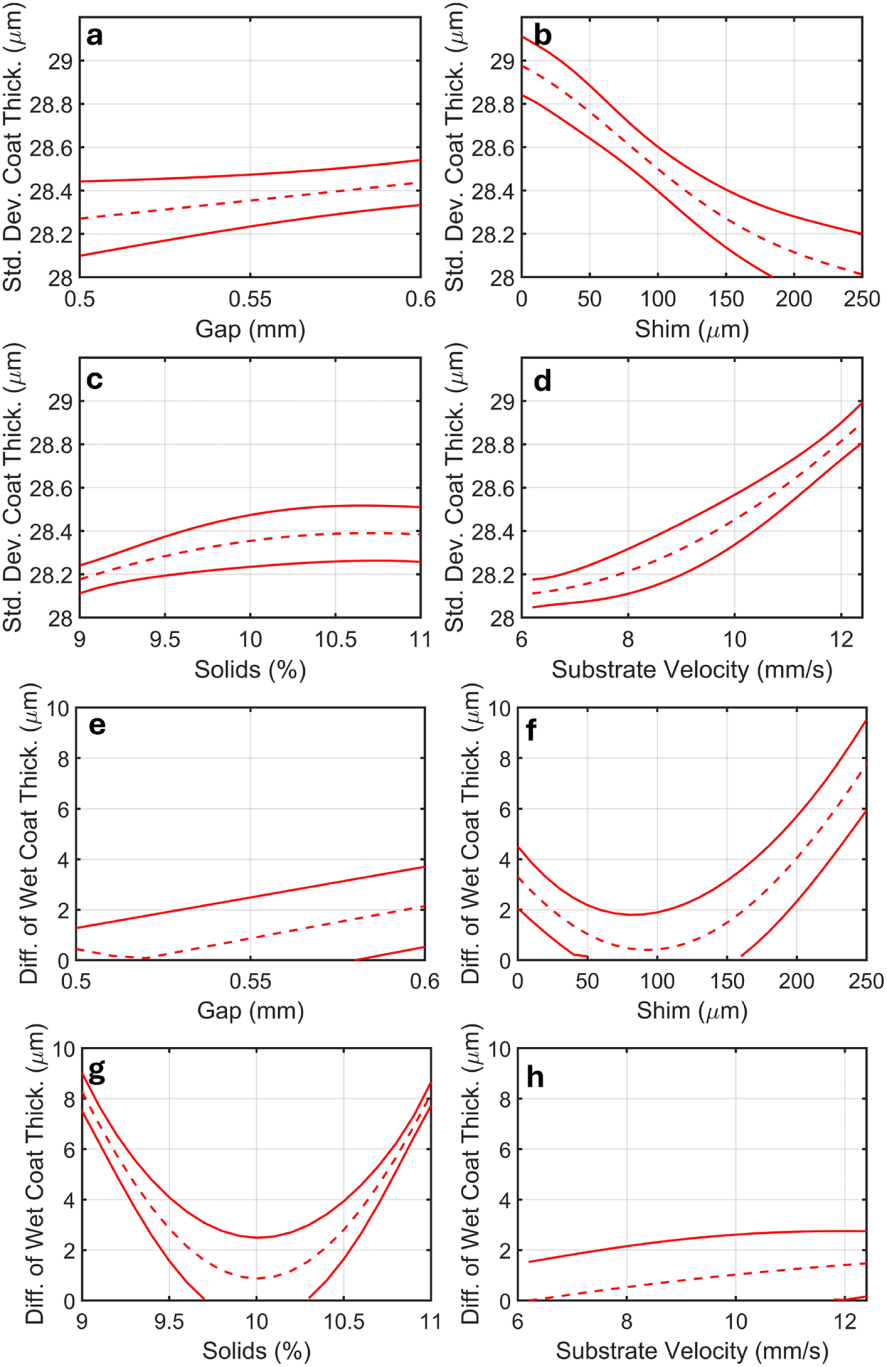
Variable effect plot of standard deviation of wet coating thickness (**a–d**) and wet coating thickness difference from the target (**e–h**). The dashed lines show the variable effect and the solid lines indicate the 95 % confidence interval. A horizontal line would indicate no effect.

Figure 6 shows a variable effect analysis of the RBFNN. This analysis is conducted by systematically varying, in turn, a single coating parameter within the surrogate model while holding all other coating parameters constant. The resulting changes in the model outputs provide valuable insights into the influence of each individual coating parameter. In this study, the fixed parameters were a coating gap of 0.55 mm, shim thickness of 130 $\upmu$m, solids of 10 % substrate velocity of 9.6 mm/s.

The coating parameters with the largest influence on coating uniformity and difference of wet coating thickness are shim thickness and coating solution solids, respectively (Fig. 6). This is further confirmed by Shapley Values, which are presented in the Supplementary Information (Figs. S7 and S8). Additionally, Shapley Values reveal that the coating gap significantly affects wet coating thickness. Shim thickness is commonly adjusted through the disassembly of the slot die and the insertion of a new shim of known thickness^42^. Some industrial coating lines have the ability to adjust the solids content inline, but many require the coating solution to be reformulated afresh using a batch process^1^. This highlights the power of the RBFNN-RVEA method, as these influential parameters, are slow to investigate experimentally, increasing the time taken for the traditional trial and error optimization of the coating, for these crucial parameters.

### Comparison of RBFNN-RVEA predictions with coating theory

Schmitt et al. investigated how coating uniformity varied within the coating window and termed regions with better coating uniformity the “quality window”^10^. The authors found that a lower dimensionless gap and lower Capillary number gave better coating uniformity. The dimensionless gap, $h^*$, and Capillary number, *Ca*, are defined below;

2 $$\begin{aligned} h^* = h/t \end{aligned}$$

Where *h* is coating gap, and *t* is wet coating thickness.

3 $$\begin{aligned} Ca = \frac{\eta . U}{\sigma } \end{aligned}$$

Where is $\eta$ is coating solution viscosity, *U* is substrate velocity and $\sigma$ is surface tension.

**Fig. 7:**
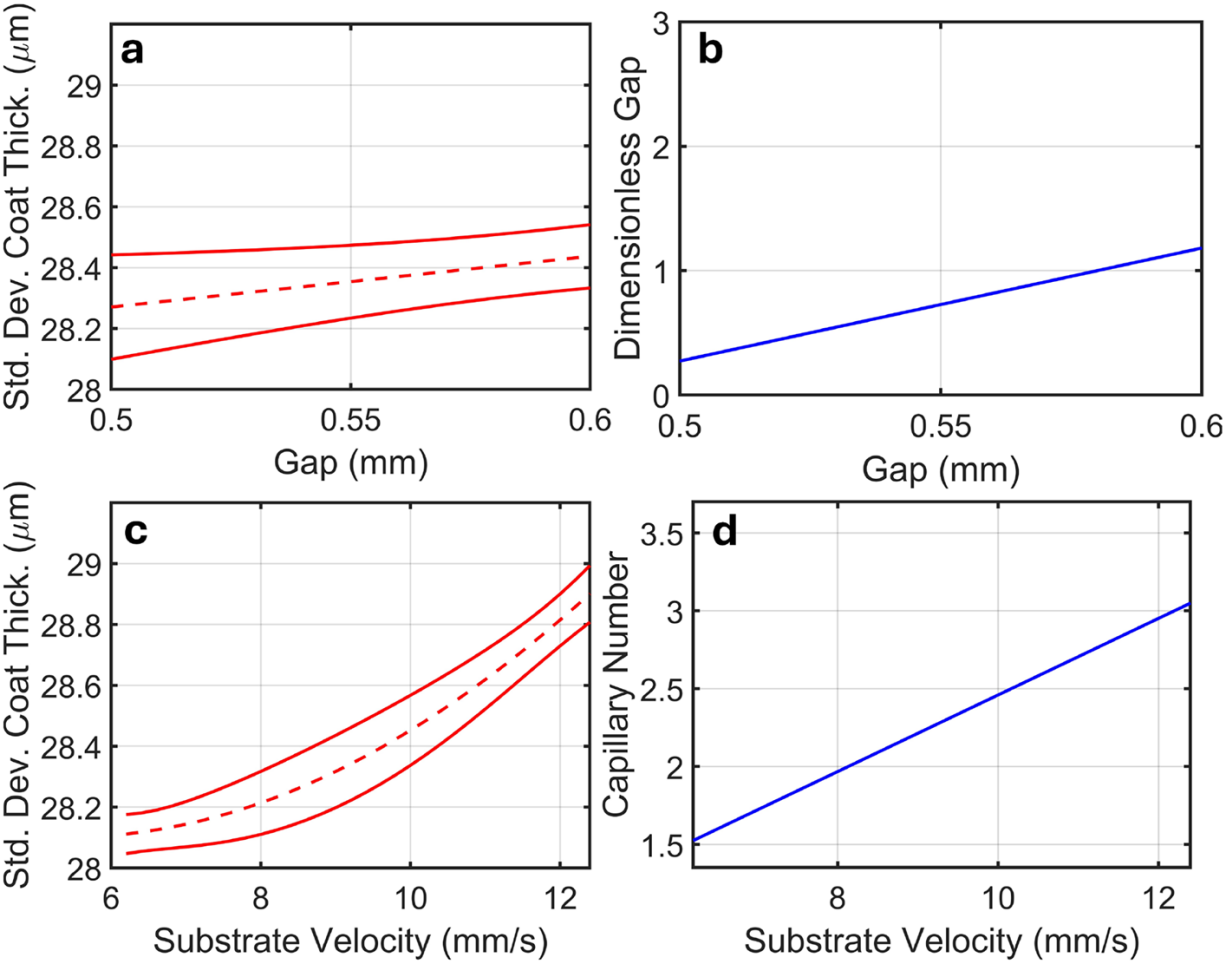
(**a**) Variable effect plot of coating gap on coating uniformity (red) and (**b**) the effect of coating gap on dimensionless gap (blue). (**c**) Variable effect plot of substrate velocity on coating uniformity (red) and (**d**) the effect of substrate velocity on Capillary number (blue).

The RBFNN-RVEA method suggests using the lowest gap possible in order to achieve better coating uniformity, as shown in Figs. 5 and 6a. This is in agreement with Schmitt’s observations, that a lower dimensionless gap gives better coating uniformity^10^. Indeed, the dimensionless gap can be mapped onto the RBFNN variable effect analysis as a predictor of change in coating uniformity, as shown in Fig. 7. It’s worth noting that the meniscus guide protrudes the slot die by 0.47 mm so the gap of 0.50 mm positions the slot die as close to the substrate before contact between the meniscus guide and substrate occurs. This highlights the RBFNN’s ability to predict the effect of changing coating parameters on coating uniformity.

Additionally, the RBFNN-RVEA method suggests the use low substrate velocities to achieve optimal coating uniformity, as shown in Figs. 5 and Fig. 6d. As substrate velocity is directly proportional to Capillary number (Eq. 3), this again is in agreement with Schmitt’s observations that a lower capillary number gives a better coating uniformity. Figure 7 shows the RBFNN predictions on the effect of substrate velocity on coating uniformity, mapped onto the Capillary number, highlighting the relationship between fluid dynamics and coating process outcomes.

The shim and solids are more challenging to map onto Capillary number, as the effect of these variables on shear stress and therefore the viscosity of the non-Newtonian coating solution, is challenging to accurately predict. Additionally, mapping the difference of wet coating thickness onto dimensionless fluid properties is not well explored in the literature.

### Experimental validation

**Fig. 8:**
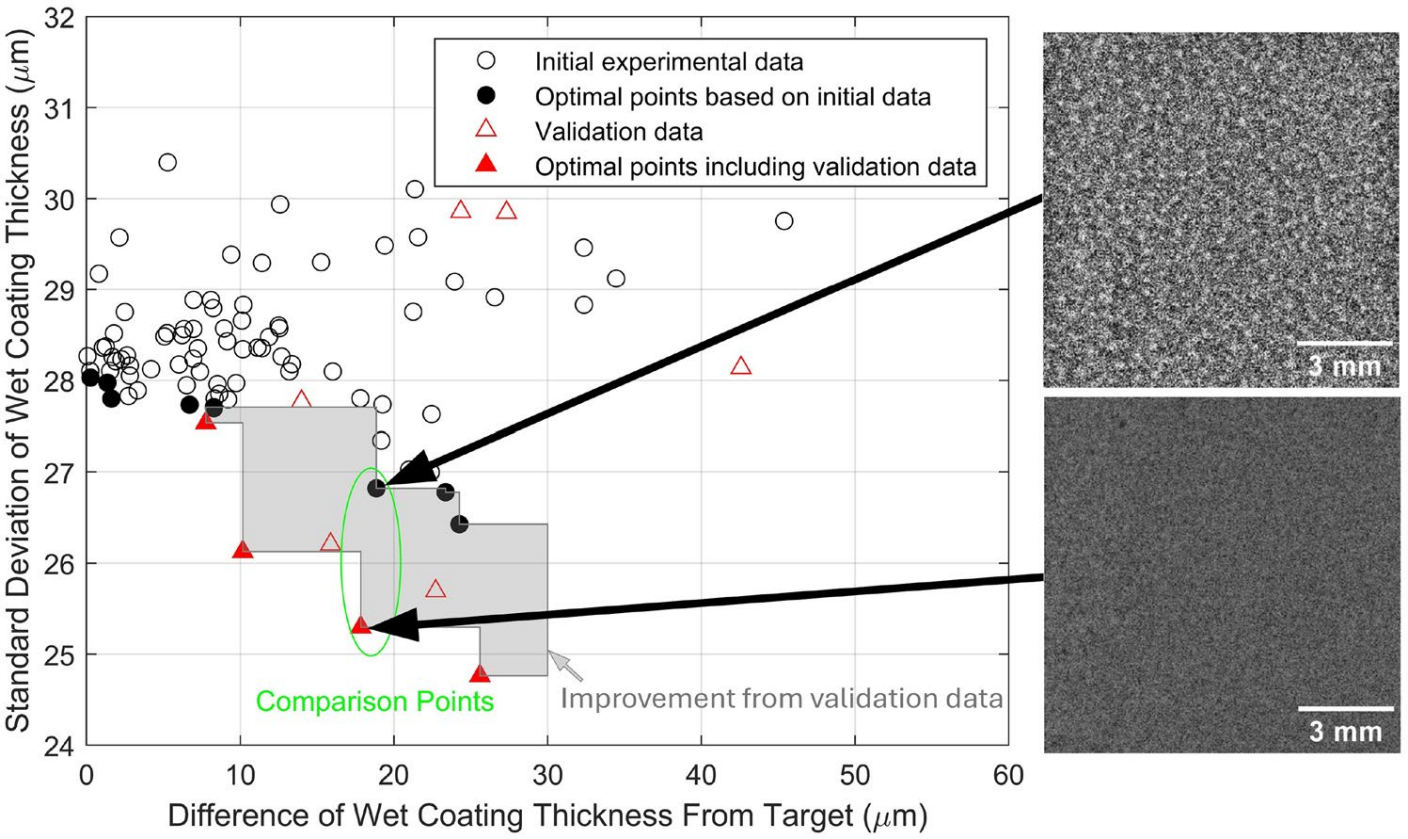
Left hand side; graph displaying the validation data overlaid on the initial experimental data, with the grey shaded region highlighting the improvement upon inclusion of the validation data. Right hand side; Cropped transmission images of a coating from the initial experimental data (upper right) and from the validation data (lower right) to highlight the improvement in coating uniformity with using RVEA predicted parameter sets. An adaptive histogram equalization has been performed to the cropped images. The unprocessed images are displayed in Supplementary Information Fig. S6.

To validate the overall optimization framework, ’unseen’ (by the machine learning training process) experiments were produced. Ten equally in position along the Pareto front spaced parameter sets were selected and tested experimentally to explore the models’ generalization ability. The positions of these ten points is shown in Supplementary Information Fig. S4. The new coating property measurements at these ten RVEA predicted Pareto front positions are shown in Fig. 8 and a comparison between one initial experimental data point and one point from the validation data is shown in Table 1.

**Table 1:** Comparison of process inputs and features of the coating from optimal points in the initial experimental data and validation data. The selected points are shown in Fig. 8.

Data source	Gap (mm)	Velocity (mm/s)	Solids (%)	Shim (µm)	Diff. of wet coating thickness from target (µm)	Std. dev. of wet coating thickness (µm)
Initial experimental data	0.55	9.3	9.0	250	18.86	26.82
RVEA validation data	0.50	7.8	9.1	250	17.84	25.30

Out of the ten validation points, six resulted in improved properties compared to the initial experimental data. Among these, one parameter set from the Pareto front of the RBFNN model, when experimentally validated, achieved the lowest coating uniformity recorded, representing a substantial improvement over the initial data. Infact, the five coatings with the best coating uniformity, are all from the RVEA validation data. This result highlights a significant enhancement in the coating’s characteristics, potentially unlocking substantial performance gains if integrated into a device^15^, such as a Li-ion battery^1,16^ or organic photovoltaic device^18^. Furthermore, eight of the ten RVEA validation data sets ranked within the top 41 % of the experimental data, in terms of coating uniformity, highlighting the effectiveness of this approach.

Hyper-volume analysis is a common method to measure the objective space covered by a set of solutions; the larger the hyper-volume, the better the solutions that have been achieved with experimental results^43,44^. The hyper-volume analysis is improved from 0.68 to 0.84 upon the inclusion of the validation data. This supports the determination that the RVEA predicted validation points gave a significant improvement over the initial experimental data.

Figure 8 shows a comparison of an apparently close to optimal data point within the initial experimental data and a new data point suggested by the RVEA optimization. Both data points had a similar difference in wet coating thickness, but the validation data coating had a lower standard deviation of coating thickness. In the optical transmission images in Fig. 8, the coating conditions suggested by RVEA optimization gave a more uniform coating with less of a speckled thickness variation. The process conditions used in the initial experimental data comparison point and RBFNN-RVEA suggested parameter set are similar, but small changes in substrate velocity, coating gap and solids content gave the observed improvement in coating uniformity (Table 1). The multiple small changes to variables suggested by the RVEA are unlikely to be made by operators, therefore highlighting the utility of the machine learning optimization.

Some prediction inaccuracies were noted. For example, the validation data did not produce any results with a difference in thickness of less than 5 $\upmu$m, despite some of the parameters tested being predicted to be in this region. The small coating width of 10 mm on our experimental rig, increases the sensitivity of the coating thickness to small changes in width, which may have hampered model accuracy. Wider coatings are typically used in industry. We note that there is also higher relative and absolute experimental error in the thickness difference compared to standard deviation of coating uniformity (see Supplementary Information Fig. S5). The 95 % confidence interval for wet coating thickness is 5.39 $\upmu$m (4.9 %) compared to 0.38 $\upmu$m (1.36 %) for coating uniformity. Pragmatically we feel that the coating performance improvements provided from only an additional ten data points would be of considerable benefit to real world process optimization. In addition, we would not expect an operator to instigate such nuanced parameter changes, and feel the “best” coating achieved by our machine learning assisted method would out perform existing empirical approaches. With more extensive industrial training data, the model accuracy would be expected to increase, potentially leading to more accurate modeling in this challenging output region.

## Conclusion

This study developed a machine learning framework to optimize roll-to-roll slot die coating, using a Radial Basis Function Neural Network trained on initial experimental data. The model achieved high predictive accuracy, with mean absolute error values below 11.5 %. Sensitivity analysis and Pareto front exploration identified shim thickness and substrate velocity as the most influential parameters on coating uniformity. Mapping predictions to coating theory revealed that improved uniformity aligns with lower dimensionless gap and lower Capillary number, consistent with prior literature^10^. A reference vector evolutionary algorithm proposed optimized parameter sets, targeting improved coating properties, which were experimentally validated. These yielded a significant increase in hypervolume fraction from 0.68 to 0.84 and produced the five most uniform coatings in the study. Compared to traditional trial and error optimization approaches typically implemented in roll-to-roll slot die coating, the surrogate model assisted optimization offers increased understanding of slot die coating behavior alongside providing rapid and large improvements in coating properties. Coating thickness predictions were less reliable, likely due to the narrow 10 mm width used in experiments, increasing the sensitivity of coating thickness to variations in width. However, industrial-scale widths (>300 mm) and precision equipment are expected to mitigate this issue. The small computation cost of less than 1 h, coupled with the short experimental duration of 2 h means that this method can feasibly be implemented to industrial lines in real time. Further enhancements in machine learning modeling could be achieved with larger and more diverse training data sets. Employing experimental design techniques such as Latin Hypercube Sampling (LHS) can facilitate more efficient and representative exploration of multivariate parameter spaces, thus maximizing information gain per experiment. Furthermore, incorporating replicate (repeat) measurements at selected parameter settings would enable more accurate assessment of process and measurement variability, as well as improve model reliability by directly accounting for experimental noise. Additional coating properties, such as edge elevations, edge position, or alternative uniformity metrics, and different coating solutions could also be incorporated into machine learning models, provided that reliable metrology is available. Moreover, integrating additional parameters, such as substrate pre-treatment, surfactant concentration, and slot die manifold design into the algorithm and employing physics-informed machine learning based on fundamental slot die coating principles may offer deeper insights into complex coating behaviors and improve model generalizability. We aim for this study to serve as an initial step toward the broader integration of machine learning guidance and a metrology- and data-driven approach to optimizing slot-die coating. Considering the critical role of coated products in meeting societal energy storage and generation demands, we hope this work encourages further research in this domain.

## Electronic supplementary material

Below is the link to the electronic supplementary material.

Supplementary Material 1

## Data Availability

Data sets generated during the current study are available from the corresponding author on reasonable request.
