# Supplementary material for: Surrogate-assisted optimization of roll-to-roll slot die coating
Source: Sci Rep. 2025 Aug 9;15:29185. doi: 10.1038/s41598-025-11279-1 (PMC12335566; doi:10.1038/s41598-025-11279-1)
Supplement: Supplementary file 1 — Supplementary Material 1 [file 41598_2025_11279_MOESM1_ESM.pdf]

# Supplementary Information For Surrogate-Assisted Optimization of Roll-to-Roll Slot Die Coating

**Christopher Passmore[1], Kai E. Wu[2], Jonathan R. Howse[1], George Panoutsos[2], and Stephen J. Ebbens[1,*]**

[1]School of Chemical Materials and Biological Engineering, The University of Sheffield, Sheffield, S1 3JD, United Kingdom
[2]School of Electrical and Electronic Engineering, The University of Sheffield, Sheffield, S1 4WD, United Kingdom
[*]s.ebbens@sheffield.ac.uk

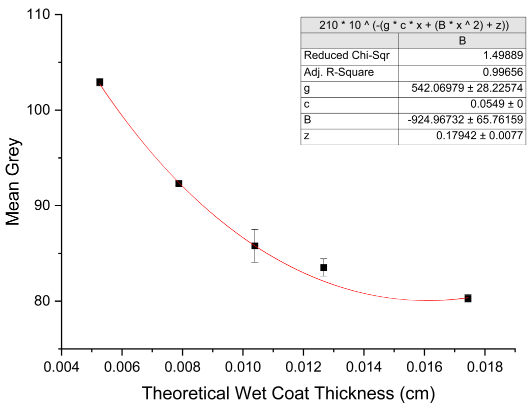


**Figure S1.** Mean Grey to thickness calibration graph with fitting based on extended Beer-Lambert Law

The Beer-Lambert law relies on several assumptions (such as the sample being thin, dilute and not reflecting light), which are not held for this application. Therefore, it is necessary to extend the law in order to account for these phenomenon. Calibration samples were run at the same conditions for 12 minutes and data from the second half of each run was analyzed. If the pump rate, substrate velocity, and coating width (from image analysis) is known, the wet coating thickness can be extracted. This is how the wet coating thickness can be determined for these calibration points in Figure S1. The RVEA optimization targeted a “mean grey” value of 85.5 which corresponds to a wet coating thickness of 108.7 $\mu$m.

For cross-validation, MAE and RMSE are calculated using the equations below;

$$MAE = \left| \frac{\sum_{n}^{i=1} y_i - \hat{y}_i}{n} \right| \tag{1}$$

$$RMSE = \sqrt{\frac{1}{n}\sum_{i=1}^{n}(y_i - \hat{y}_i)^2} \tag{2}$$

Using a higher substrate velocity increases the output of a given production line per unit time[1]. The intrinsic limit on the speed that a slot die coating production line can be operated at is called the “maximum wetting speed”[1]. Beyond this speed, dynamic wetting failure occurs; the coating solution cannot adhere to the substrate and beads form rather than a continuous thin film. This phenomenon is well studied[2,3], but the effect of substrate velocity on coating uniformity within the operating window has not been fully elucidated. Figure S3 shows the predicted effect on the Pareto front when the parameter sets are limited to using only the highest substrate velocity achievable on the roll-to-roll slot die coater. For a given difference in wet coating thickness, the coating uniformity is significantly worse when limited to choosing the highest substrate velocity. A possible explanation for this effect is that at higher coating speeds, the coating is subjected to more shear forces when exiting the slot die. These shear forces may significantly disrupt the flow of coating solution exiting the slot die and hence increase the standard

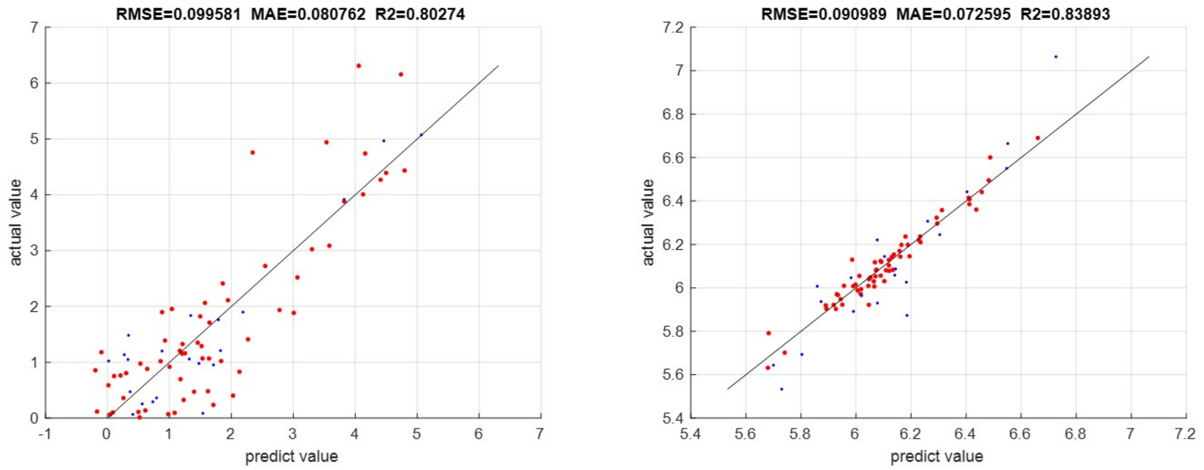


**Figure S2.** RBFNN cross validation of **a** Difference of wet coating thickness from target and **b** Standard Deviation of Wet Coating Thickness.

deviation of coating thickness. Therefore, the RBFNN model allows operators to make informed decisions about the advantages and trade-offs of using a higher line speed; the operator has to decide between a higher output or an improved coating quality.

**Table 1.** Comparison of hyper-volume analysis of the initial experimental data and the same data set, including the RVEA validation data.

| Data Source | Hyper-Volume |
|---|---|
| Initial Experimental Data | 0.68 |
| Including RVEA Validation Data | 0.84 |

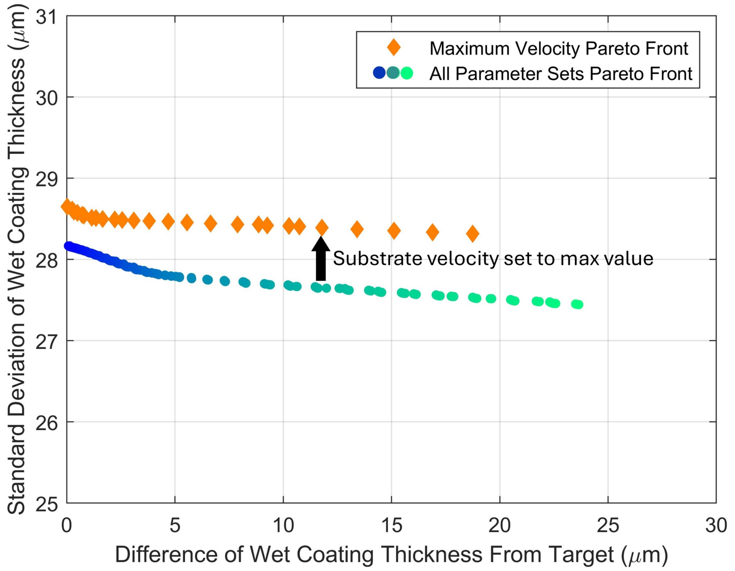


**Figure S3.** Graph showing the effect of limiting substrate velocity to the highest speed on the RFBNN predicted Pareto front.

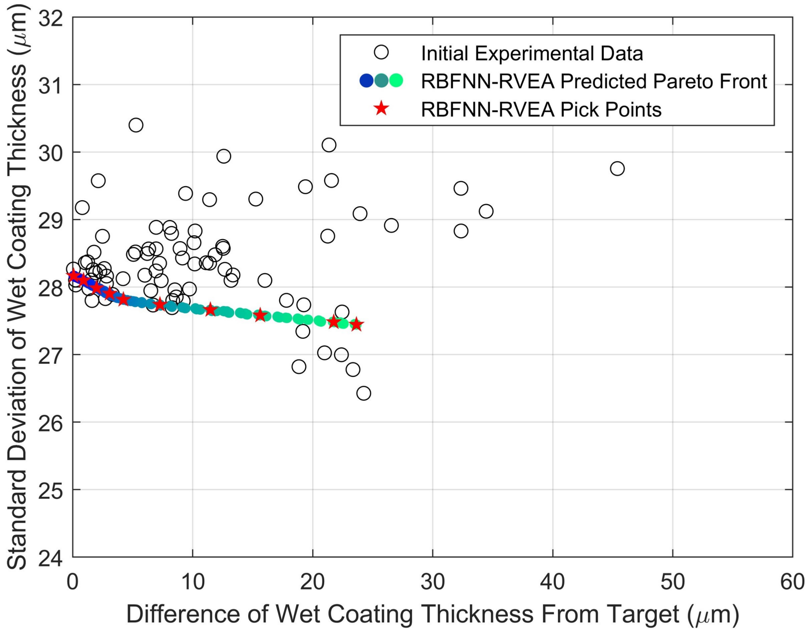


ht

**Figure S4.** Graph showing training data with RBFNN-RVEA predicted Pareto front with the selected ten validation points overlaid

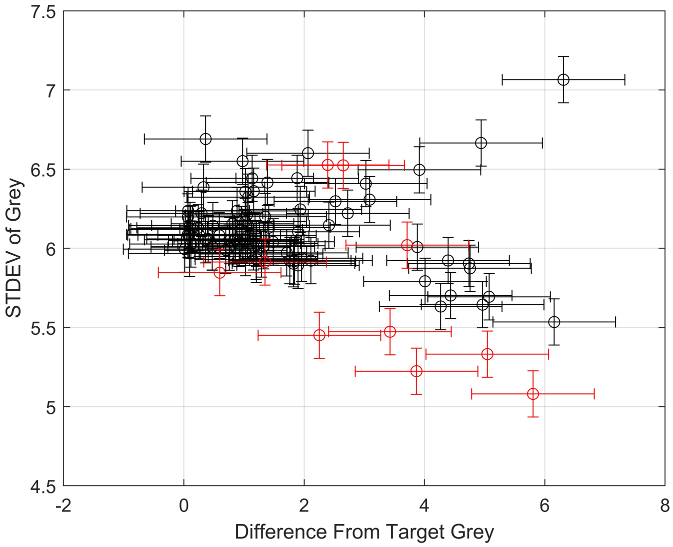


**Figure S5.** Graph showing data used in this article, with error bars shown. The axis are in dimensionless grey values rather than thickness.

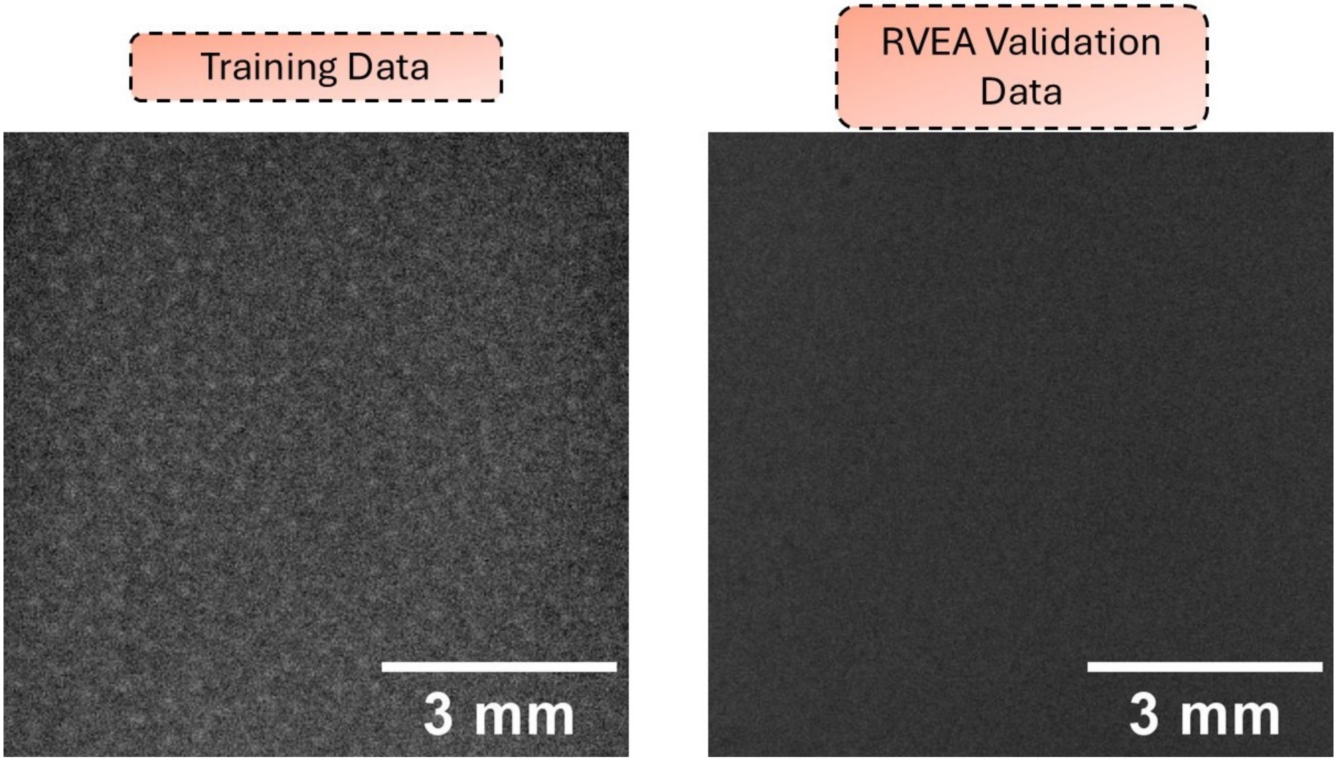


**Figure S6.** Unprocessed images of coatings highlighted in validation data figure.

## Gaussian Processing and Surrogate Vector Regression Modeling

The SVR regression model implemented here is designed for a 4-input, 2-output dataset, utilizing a subsetting approach to enhance robustness and evaluate performance. Specifically, the model is trained and tested across 10 iterations, where each iteration randomly selects 70 % of the dataset (81 samples) for training and reserves the remaining 30 % for testing. This random subsetting, achieved using MATLAB's randperm function with a fixed random seed (rng(1)) for reproducibility, ensures diverse training and testing splits, reducing overfitting and providing a reliable estimate of generalization performance. For each iteration, two separate Support Vector Machine (SVM) models with Gaussian kernels are trained—one for each output—using standardized inputs, an epsilon value of 0.1, and automatic kernel scale optimization. Error metrics (RMSE, MAE, R-squared) are computed for both training and testing sets, and their means and standard deviations are reported in absolute and percentage forms, normalized by the target variable's range. This subsetting strategy, combined with the Gaussian kernel's ability to capture non-linear relationships, enables the model to effectively learn complex patterns while providing comprehensive performance insights across multiple data splits.

**Table 2.** Error analysis of SVR Model for Difference of Wet Coating Thickness.

| Data Set | RMSE (%) | MAE (%) | $R^2$ (%) |
|---|---|---|---|
| Training | 19.73 | 12.49 | 30.98 |
| Testing | 23.68 | 16.06 | 8.38 |

**Table 3.** Error analysis of SVR Model for Standard Deviation of Wet Coating Thickness.

| Data Set | RMSE (%) | MAE (%) | $R^2$ (%) |
|---|---|---|---|
| Training | 10.86 | 8.09 | 50.40 |
| Testing | 13.24 | 10.06 | 32.16 |

The Gaussian Process Regression (GP) model implemented for the 4-input, 2-output dataset employs a subsetting approach to ensure robust training and reliable performance evaluation. The dataset, consisting of 81 samples, is divided into 10 distinct iterations, with each iteration randomly selecting 70 % of the data for training and reserving the remaining 30 % for testing. This random subsetting is facilitated by MATLAB's randperm function, with a fixed random seed (rng(1)) to ensure reproducibility across runs. For each iteration, two separate GP models—one for each output—are trained using a squared exponential kernel, standardized inputs, and automatic hyperparameter optimization limited to 30 evaluations for computational efficiency. The random splits help mitigate overfitting and provide a comprehensive assessment of the model's generalization capability by exposing it to varied subsets of the data. Error metrics (RMSE, MAE, R-squared) are calculated for both training and testing sets, with means and standard deviations reported in both absolute units and percentages (normalized by the target variable's range). This subsetting strategy, paired with GP's probabilistic framework, allows the model to capture complex, non-linear relationships while offering robust performance insights across diverse data partitions.

**Table 4.** Error analysis of GP Model for Difference of Wet Coating Thickness.

| Data Set | RMSE (%) | MAE (%) | $R^2$ (%) |
|---|---|---|---|
| Training | 3.91 | 3.02 | 95.20 |
| Testing | 17.28 | 12.60 | 40.56 |

**Table 5.** Error analysis of GP Model for Standard Deviation of Wet Coating Thickness.

| Data Set | RMSE (%) | MAE (%) | $R^2$ (%) |
|---|---|---|---|
| Training | 1.16 | 0.86 | 99.17 |
| Testing | 10.67 | 7.74 | 55.15 |

## Shapley Values

Shapley values are a concept from cooperative game theory that have been adapted to interpret machine learning models. In this context, they help explain how much each coating parameter contributes to a model's output. For each prediction, Shapley values fairly distribute the "credit" or "blame" for the prediction among the features, considering all possible combinations of features. The Shapley values for this RBFNN method were extracted and are displayed in S7 and S8.

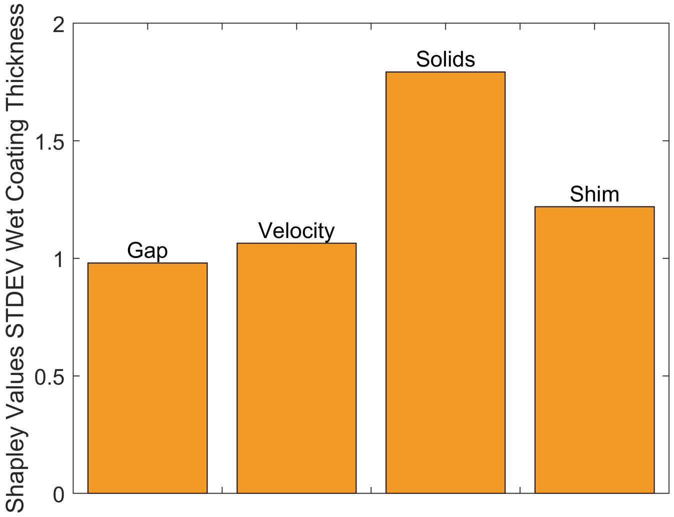


**Figure S7.** Shapley Values of Standard Deviation of Wet Coating Thickness.

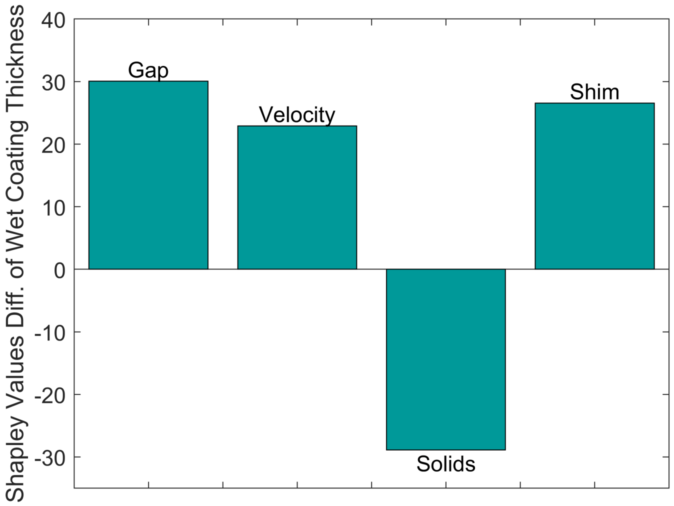


**Figure S8.** Shapley Values of Difference of Wet Coating Thickness from Target.

## References

1. Ding, X., Liu, J. & Harris, T. A. L. A review of the operating limits in slot die coating processes. *AIChE J.* **62**, 2508–2524, DOI: https://doi.org/10.1002/aic.15268 (2016). https://aiche.onlinelibrary.wiley.com/doi/pdf/10.1002/aic.15268.

2. Chin, C.-P., Wu, H.-S. & Wang, S. S. Improved coating window for slot coating. *Ind. & Eng. Chem. Res.* **49**, 3802–3809, DOI: 10.1021/ie801900t (2010). https://doi.org/10.1021/ie801900t.

3. Jakubka, F. *et al.* Determining the coating speed limitations for organic photovoltaic inks. *Sol. Energy Mater. Sol. Cells* **109**, 120–125, DOI: https://doi.org/10.1016/j.solmat.2012.10.007 (2013).
